# Factors Associated with the Health-Related Self-Care Capacity of Overweight and Obese People

**DOI:** 10.3390/healthcare12121226

**Published:** 2024-06-20

**Authors:** Liz K. Tenorio-Guerrero, Romara Puente-Alejos, Janett V. Chavez Sosa, Edda E. Newball-Noriega, Salomon Huancahuire-Vega

**Affiliations:** 1 Human Medicine School, Universidad Peruana Unión (UPeU), Lima 15464, Peru; liztenorio@upeu.edu.pe (L.K.T.-G.); romarapuente@upeu.edu.pe (R.P.-A.); salomonhuancahuire@upeu.edu.pe (S.H.-V.); 2 Posgraduate School, Universidad Peruana Unión (UPeU), Lima 15464, Peru; janett.chavez@upn.pe; 3 General Directorate of Research, Universidad Peruana Unión (UPeU), Lima 15464, Peru

**Keywords:** beliefs, health-related self-care, overweight, obesity, adult population

## Abstract

This study aimed to investigate the relationship between beliefs about obese people and health-related self-care among overweight and obese people, considering sociodemographic aspects. This study adopted a cross-sectional design. The sample consisted of 207 participants selected through a simple random sampling method. The “Beliefs About Obese Persons Scale” (BAOP) and the “Self-Care Agency Rating Scale-Revised” (ASA-R) questionnaires were applied to data collection. The results showed that 82.6% believed that obesity is a condition the individual cannot control, and 74.4% expressed inadequate self-care regarding their health. A multivariate analysis found that belonging to the adult age group increases the probability of presenting adequate health-related self-care by 4.7 times (95% CI = 1.892–11.790) compared to older adults. The belief that obesity is an uncontrollable condition increases the probability of inadequate self-care by 6.3 times (95% CI = 2.360–16.924), in contrast to the perception that it is a controllable condition. Moreover, overweight people are 0.139 times (95% CI = 0.044–0.443) less likely to have adequate self-care compared to people with obesity. In conclusion, being an adult and having the belief that obesity is a condition that can be controlled is associated with adequate health-related self-care, while being overweight is associated with inadequate health care.

## 1. Introduction

Obesity has emerged as a global public health challenge, acquiring epidemic proportions in recent decades [1]. In 2022, 2.5 billion adults aged 18 years and older were overweight, including over 890 million adults who were living with obesity, with an associated mortality estimated at 2.8 million people [2]. In South America, obesity has become a serious health problem [3]. In adults, rates vary from 23.7% in Uruguay to 34.4% in Chile [4]. The prevalence of obesity in Brazil increased significantly between 2006 and 2019 with an annual increase of 2% [5]. In Argentina, there is a high rate of excess weight in the young population; more than 30% of adolescents between 13 and 15 years old are obese and overweight [6]. In Peru, data from the Demographic and Family Health Survey (ENDES) of the National Institute of Statistics and Informatics (INEI) reveal that approximately 23.7% of Peruvian adults suffer from obesity; in comparison, 39.7% are overweight [7].

Obesity is characterized by excess body fat that carries significant health risks, such as type 2 diabetes, cardiovascular disease, hypertension, and certain types of cancer [8]. However, beyond medical implications, obesity is also surrounded by a social stigma that can have negative effects on the lives of those affected [9].

Health-related self-care is a fundamental part of managing obesity and related diseases. It comprises a series of behaviors and decisions people make to maintain and improve their health, including healthy eating, regular physical activity, managing stress, and seeking appropriate medical care [10]. However, the stigma associated with obesity can act as a barrier to effective health-related self-care, as it can influence self-esteem, motivation, and willingness to adopt healthy behaviors [11].

Moreover, beliefs and attitudes towards people with obesity play a crucial role in perpetuating this stigma. Negative perceptions, stereotypes, and discrimination towards people with obesity can influence their emotional, social, and physical well-being, as well as their ability to access and participate in health-related self-care. This stigma can manifest in different settings, including the media, the workplace, the medical field, and everyday social interactions [12].

A study in Great Britain showed that belief in environmental or genetic influence on overweight was linked to more support for government obesity policies [13]. In comparison, genetic attributions were related to greater support for free weight-loss treatments and healthy lifestyle promotional campaigns. In Australia, disgust was the main predictor of negative attitudes towards obese people, mediating the relationship between perceptions of control and attitudes [14]. Obese people were rated less favorably and were perceived to be more repulsive than other social groups, with disgust mediating the relationship between perceived control and favorability [13,14].

Due to these considerations, this study aimed to determine the relationship between beliefs about people with obesity and health-related self-care in overweight and obese people, considering sociodemographic aspects.

## 2. Methods

### 2.1. Type of Study, Population, and Sample

The study had a non-experimental, cross-sectional, and analytical design. Research was carried out in the district of Los Molinos, located in the province of Ica, Peru. The population is mainly distributed in small rural communities scattered throughout the territory. The district’s economy is mainly based on agriculture. Although the district is mainly rural, it has basic services such as drinking water, electricity, and public transportation.

According to the latest available census, the district has a population of 6987 inhabitants, of which 1397 suffer from overweight and obesity. The sample was selected using a simple random probabilistic sampling method, calculating a sample size of 207 inhabitants with a confidence level of 95%, a margin of error of 5%, and a proportion of 0.8 (80% prevalence of inappropriate health-related self-care).

**Figure 1:**
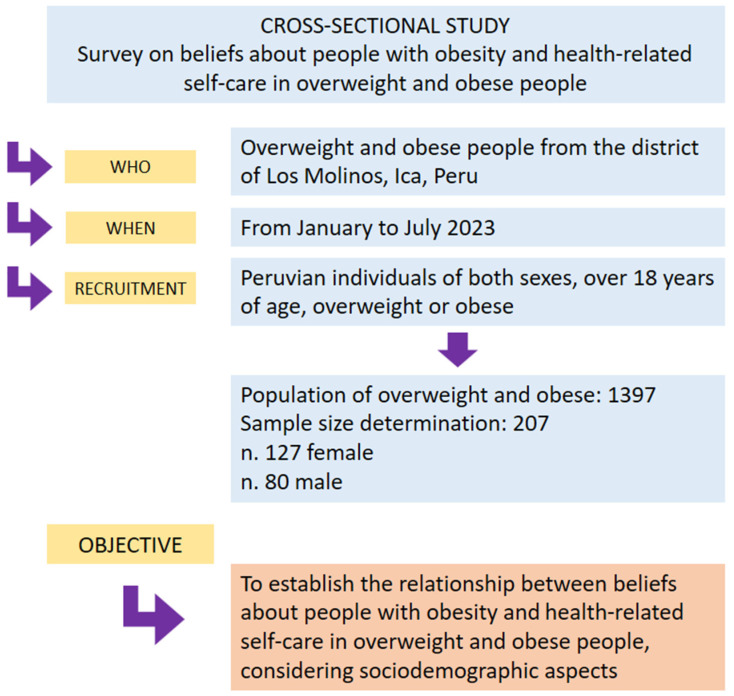
Study design, including data collection, participant recruitment, and objective.

The inclusion criteria for the participants were that they were Peruvian residents of both sexes, over 18 years of age, overweight or obese, and who agreed to participate in the study. Foreign residents, minors, pregnant women, those with an average body mass index (BMI), and those who did not sign the informed consent form were excluded. Participants were recruited through home visits conducted by a team of trained interviewers. A detailed explanation of the study was provided to each potential participant, ensuring understanding of the study’s objectives and procedures. For participants who could not read, the informed consent form was read aloud in the presence of an independent witness who verified and certified that the participant understood the information provided (Figure 1).

### 2.2. Data Collection Instruments and Techniques

For data collection, a survey technique and questionnaire instrument were used. For the variable beliefs about people with obesity, the Beliefs About Obese Persons Scale (BAOP) was used, translated, and validated into Spanish, with a reliability of Cronbach’s Alpha greater than 0.6 [15]. It consists of eight statements that directly address the possible causes of obesity. Participants must express their level of agreement with each statement using a four-point Likert scale, ranging from 1 (completely disagree) to 4 (completely agree). The total score is calculated by summing the responses for each item; a score greater than 30 indicates the view that obesity is a condition that individuals can control.

For the self-care variable, the Self-Care Agency Rating Scale-Revised (ASA-R) was used, translated, and validated into Spanish in the Colombian population, with a reliability of Cronbach’s Alpha of 0.79 [16]. The instrument is unidimensional and consists of eight items that evaluate whether people have and develop self-care capacity. It is measured on a Likert scale with alternatives ranging from totally disagree [1] to totally agree [5]. The final scoring scale classifies self-care as adequate (32–40 points) and inadequate (<32 points).

Additionally, sociodemographic data were collected, such as sex, age, level of education, marital status, employment status, people living in the home, economic income, and body mass index.

### 2.3. Data Analysis

The statistical package SPSS v.24 was used for data analysis and processing. Simple frequency tables were used for categorical variables in univariate analysis. For the bivariate analysis, contingency tables and the Chi-square test were used. The variables that had a statistically significant relationship had a *p*-value < 0.05. Finally, binary logistic regression was performed for the multivariate analysis, which considered health-related self-care as the dependent variable and sociodemographic characteristics and beliefs about people with obesity as independent variables.

### 2.4. Ethical Aspects

The Faculty of Health Sciences of the Universidad Peruana Union ethics committee approved the work (RS 2023-CE-FCS-UPeU-030). In addition, informed consent was signed prior to completing the questionnaires. In the cases of participants who were unable to sign, a fingerprint with a witness signature was used to confirm their agreement.

## 3. Results

Of the 207 people surveyed, 61.4% were women, and 38.6% were men. Most of the participants belonged to the adult age group (76.3%). Furthermore, 66.2% had a secondary education level, 82.6% were married or cohabiting, 77.8% had a job, and 71% reported a monthly income greater than 1025 soles (minimum wage in Peru). In turn, 59.4% indicated that 1 to 4 people lived in their home. In total, 76.3% of the participants were overweight (Table 1).

Regarding the study variables, 82.6% of people believed that obesity is a condition that the individual cannot control, while 17.4% perceived obesity as a condition that can be controlled. On the other hand, 74.4% expressed inadequate self-care of their health, and 25.6% expressed adequate self-care of their health (Table 1).

In the bivariate analysis, age (*p* = 0.000), level of education (*p* = 0.000), body mass index (*p* = 0.014), and beliefs about obesity (*p* = 0.004) were significantly related to health-related self-care (Table 2).

Statistically significant variables (age, level of education, BMI, and beliefs about obesity) were included in the multivariate analysis. The multivariate analysis showed that being an adult increases the probability of having adequate health-related self-care by 4.7 times (95% CI = 1.892–11.790; *p* = 0.001) compared to older adults. Likewise, the belief that obesity is a condition that the individual cannot control increases the probability of inadequate self-care 6.3 times (95% CI = 2.360–16.924; *p* = 0.000) as opposed to people who believe it is a condition that can be controlled. On the other hand, overweight people are 0.139 times (95% CI = 0.044–0.443) less likely to perform adequate self-care compared to people with obesity (Table 3).

## 4. Discussion

The purpose of this study was to determine the relationship between beliefs about people with obesity and health-related self-care in overweight and obese people, considering sociodemographic aspects. This study showed that the majority of the overweight and obese population believed that obesity is a condition that the individual cannot control. Similarly, a study in the United States showed that the majority of those surveyed consider lifestyles to strongly influence obesity, while only a few attribute great importance to heredity [17]. In Latin American countries such as Mexico, it was observed that the vast majority had a negative attitude towards people with obesity, and that this health condition could not be controlled [18]. In Chile, overweight or obese people tended to underestimate their nutritional status, perceiving that this condition could not be controlled due to hereditary factors [19]. Likewise, those who believe in heredity as a determining factor in obesity tend to have lower levels of physical activity and consumption of fruits and vegetables. Additionally, a study of the Peruvian population showed that the majority of participants did not recognize obesity as a disease but rather as a risk factor for heart disease [20]. Many did not know its meaning or confused it with bad eating habits. In addition, frequent inconveniences in daily activities due to obesity were mentioned, and a widespread belief that heart problems had an exclusively genetic origin, regardless of body weight, was observed.

These results can be explained by the concept of “locus of control.” According to this theory, people can perceive events and outcomes as controlled by internal (e.g., individual actions and decisions) or external (e.g., environmental or genetic circumstances) factors. The fact that a large proportion of overweight and obese people perceive the condition as something they cannot control suggests an orientation toward an external locus of control about their weight [21]. This may be influenced by various factors, such as exposure to negative messages about obesity, lack of access to resources to adopt healthy behaviors, and perceived social or economic barriers to lifestyle changes. Additionally, obesity has historically been stigmatized in many societies, which can lead people to internalize negative beliefs about themselves and their ability to control their weight. The perception that obesity is an uncontrollable condition can contribute to feelings of guilt, low self-esteem, and lack of motivation to make healthy lifestyle changes [22].

The majority of the participants in this study reported inadequate self-care of their health. A study of obese diabetic patients in Australia noted that those with a body mass index (BMI) equal to or greater than 35 kg/m^2^ are less likely to achieve healthy diet and exercise goals and also tend to give less importance to recommendations related to these aspects, which contributes to an increase in obesity problems [23]. By contrast, another study highlighted that obese people can improve their self-care, health, and well-being by participating in multidisciplinary obesity clinics in rural and remote areas [24]. On the other hand, an investigation carried out on obese women revealed that knowledge about the disease, the absence of harmful habits, and reasonable health control were the most promoted aspects of physical self-care [25]. However, a low development of psychological self-care was observed, characterized by passive coping with the disease, negative emotions, and dissatisfaction with body image, which affected self-evaluation, along with a lack of vision for the future. Regarding social self-care, this was favored by a family support network and recreational activities.

This study found that being an adult significantly increased the likelihood of having adequate self-care compared to older adults. This association can be understood through the Role Transition Model of Middle Adulthood, which suggests that adults may have a greater capacity to manage their roles and responsibilities, which can positively influence their self-care [26]. These findings are consistent with previous research that has identified an association between age and health-related self-care in overweight and obese people [27].

Related to beliefs about obesity, this study found that the belief that obesity is an uncontrollable condition increased the likelihood of inadequate self-care. Similarly, previous research has identified an association between negative beliefs about obesity and lower health-related self-care [28,29]. From a theoretical perspective, this can be explained by the Health Belief Model (Health Belief Model), which postulates that beliefs about the severity of a disease and the perception of its controllability influence self-care behaviors [30].

Behavior change theory, applied to people with obesity, highlights the importance of addressing individual beliefs and attitudes to promote optimal self-care [31]. It emphasizes how modifying these beliefs and attitudes can facilitate the adoption of healthy behaviors, promote intrinsic motivation, and satisfy basic psychological needs. Furthermore, the importance of increasing confidence in people’s ability to make positive changes in their lifestyle is highlighted. These approaches offer valuable insights for designing interventions focused on strengthening positive beliefs and proactive attitudes toward self-care among people with obesity, which can lead to significant improvements in their health and well-being [11].

On the other hand, this study found that overweight people were less likely to have adequate self-care compared to people with obesity. These two groups could explain the differences in risk perception and self-care behaviors. Previous research has suggested that people with obesity may be more aware of their health status and, therefore, more motivated to engage in self-care behaviors [30,32].

Finally, people with secondary education were less likely to have adequate self-care than those with higher education. This result is consistent with previous research that has identified a significant association between educational level and health-related self-care in overweight and obese people [33]. People with a higher level of education may have better access to health information and a greater understanding of the importance of self-care, which may motivate them to engage in healthier behaviors [34].

### Limitations

Although this study provides valuable information about the relationship between obesity beliefs and health-related self-care in overweight and obese individuals, it has some important limitations. The sample used may only partially represent the target population, as it is limited to a single district in Peru, which could affect the generalizability of the findings. Furthermore, this study’s cross-sectional design makes it difficult to establish causal relationships between the variables studied. It does not allow us to examine how these relationships may change over time. Data collection based on participants’ self-reports could be subject to memory or desire for social presentation biases. Variables such as beliefs about obesity and self-care of health were measured using self-administered questionnaires, which could introduce interpretation bias. Although several variables were controlled through the multivariate analysis conducted, it is possible that other confounding factors were not considered that could influence the relationship between the variables studied. These limitations underscore the need to interpret the results cautiously and conduct future research that addresses these limitations and expands our understanding of the relationship between obesity beliefs and health-related self-care.

## 5. Conclusions

The results of this study indicate that, among overweight and obese residents, those who belonged to the older adult age group and who maintained the belief that obesity is an uncontrollable condition showed a greater probability of inadequate health-related self-care. On the contrary, people with an overweight BMI had a lower probability of adequate self-care, unlike the group with an obese BMI. These results highlight the importance of considering sociodemographic factors and beliefs about obesity in promoting health-related self-care in overweight and obese people. These findings have significant implications for designing public health interventions to improve self-care and health in this population. Recognizing that belonging to the adult age group significantly increases the likelihood of practicing adequate self-care compared to older adults underscores the need to target specific self-care interventions toward different age groups, recognizing disparities in each group’s self-care needs and capabilities. Parallel to this, understanding how the belief that obesity is an uncontrollable condition increases the likelihood of inadequate self-care highlights the urgency of addressing these negative perceptions in public health programs and in clinical interventions to promote more effective self-care among people with obesity. Furthermore, the identification that overweight people are less likely to have adequate self-care compared to people with obesity highlights the need to develop specific strategies to support this group in improving their self-care and preventing the complications of health associated with obesity.

## Figures and Tables

**Table 1:** Descriptive analysis of the sociodemographic variables and study variables.

Variables		n = 207	%
Gender	Female	127	61.4
	Male	80	38.6
Age	Adult (18–59 years)	158	76.3
	Elderly (≥60 years)	49	23.7
Educational level	No education	6	2.9
	Elementary	15	7.2
	Secondary	137	66.2
	University	49	23.7
Marital status	Single/separated/widowed	36	17.4
	Married/Co-habitant	171	82.6
Employment status	Unemployed	46	22.2
	Employed	161	77.8
People living at home	1–4 people	123	59.4
	More than 4 people	84	40.6
Economic income	Less than 1025 soles	60	29.0
	More than 1025 soles	147	71.0
Body Mass Index (BMI)	Overweight	158	76.3
	Obesity	49	23.7
Beliefs about obesity	Obesity is a condition that the individual cannot control	171	82.6
	Obesity is a condition that the individual can control	36	17.4
Health-related self-care	Inappropriate	154	74.4
	Appropriate	53	25.6

**Table 2:** Bivariate analysis according to the perception of health-related self-care in overweight and obese people.

Variables		Health-Related Self-Care				*p*-Value
		Appropriate		Inappropriate		
		n = 53	%	n = 154	%	
Gender	Female	38	71.7	89	57.8	0.073
	Male	15	28.3	65	42.2	
Age	Adult (18–59 years)	31	58.5	127	82.5	0.000 *
	Elderly (≥60 years)	22	41.5	27	17.5	
Educational level	No education	3	5.7	3	1.9	0.000 *
	Elementary	1	1.9	14	9.1	
	Secondary	23	43.4	114	74.0	
	University	26	49.1	23	14.9	
Marital status	Single/separated/widowed	6	11.3	30	19.5	0.176
	Married/Co-habitant	47	88.7	124	80.5	
Employment status	Unemployed	14	26.4	32	20.8	0.395
	Employed	39	73.6	122	79.2	
People living at home	1–4 people	30	56.6	93	60.4	0.628
	More than 4 people	23	43.4	61	39.6	
Economic income	Less than 1025 soles	14	26.4	46	29.9	0.633
	More than 1025 soles	39	73.6	108	70.1	
Body Mass Index (BMI)	Overweight	47	88.7	111	72.1	0.014 *
	Obesity	6	11.3	43	27.9	
Beliefs about obesity	Obesity is a condition that the individual cannot control	37	69.8	134	87.0	0.004 *
	Obesity is a condition that the individual can control	16	30.2	20	13.0	

* *p* < 0.05.

**Table 3:** Multivariate analysis according to the perception of health-related self-care in overweight and obese people.

Variables		OR	CI 95%		*p*-Value
			LI	LS	
Gender	Male	1			0.722
	Female	0.857	0.367	2.003	
Age	Elderly	1			0.001 *
	Adult	4.723	1.892	11.790	
Educational level	University	1			
	Secondary	0.162	0.024	1.105	0.063 *
Body Mass Index (BMI)	Obesity	1			0.001 *
	Overweight	0.139	0.044	0.443	
Beliefs about obesity	Obesity is a condition that the individual can control	1			0.000 *
	Obesity is a condition that the individual cannot control	6.321	2.360	16.924	

* *p* < 0.05; OR: odds ratio; CI: confidence intervals.

## Data Availability

The data that support the findings of this study are available on request from the corresponding author.
